# Inter- and intra-researcher reproducibility of heart rate variability parameters in three human cohorts

**DOI:** 10.1038/s41598-020-68197-7

**Published:** 2020-07-09

**Authors:** A. Plaza-Florido, J. M. A. Alcantara, J. H. Migueles, F. J. Amaro-Gahete, F. M. Acosta, J. Mora-Gonzalez, J. Sacha, F. B. Ortega

**Affiliations:** 1 PROFITH “PROmoting FITness and Health Through Physical Activity” Research Group, Sport and Health University Research Institute (iMUDS), Department of Physical and Sports Education, Faculty of Sport Sciences, University of Granada, Granada, Spain; 2 EFFECTS-262 Research Group, Department of Physiology, School of Medicine, University of Granada, 18071 Granada, Spain; 3 College of Health and Human Services, University of North Carolina at Charlotte, Charlotte, NC USA; 4 Faculty of Physical Education and Physiotherapy, Opole University of Technology, Opole, Poland; 5 Department of Cardiology, University Hospital in Opole, University of Opole, Opole, Poland

**Keywords:** Cardiology, Risk factors

## Abstract

Heart rate variability (HRV) is a valid and non-invasive indicator of cardiac autonomic nervous system functioning. Short-term HRV recordings (e.g., 10 min long) produce data that usually is manually processed. Researcher subjective decision-making on data processing could produce inter- or intra-researcher differences whose magnitude has not been previously quantified in three independent human cohorts. This study examines the inter- and intra-researcher reproducibility of HRV parameters (i.e., the influence of R–R interval selection by different researchers and by the same researcher in different moments on the quantification of HRV parameters, respectively) derived from short-term recordings in a cohort of children with overweight/obesity, young adults and middle-age adults. Participants were recruited from 3 different studies: 107 children (10.03 ± 1.13 years, 58% male), 132 young adults (22.22 ± 2.20 years, 33% males) and 73 middle-aged adults (53.62 ± 5.18 years, 48% males). HRV was measured using a Polar RS800CX heart rate monitor. The intraclass correlation coefficient (ICC) ranged from 0.703 to 0.989 and from 0.950 to 0.998 for inter-and intra-researcher reproducibility, respectively. Limits of agreement for HRV parameters were higher for the inter-researcher processing compared with the intra-researcher processing. On average, the intra-researcher differences were 31%, 62%, and 80% smaller than the inter-researchers differences based on Coefficient of Variation in children, young and middle-aged adults, respectively. Our study provides the quantification of the inter-researcher and intra-researcher differences in three independent human cohorts, which could elicit some clinical relevant differences for HRV parameters. Based on our findings, we recommend the HRV data signal processing to be performed always by the same trained researcher and we postulate a development of algorithms for an automatic ECG selection.

## Introduction

Heart rate variability (HRV) is a valid and non-invasive indicator of cardiac autonomic nervous system functioning^1,2^. Both autonomic branches (i.e., the parasympathetic and sympathetic nervous systems) are considered the main determinants of the magnitude of HRV^3^. However, from an integrative physiological point of view, the HRV has been considered an indicator of the complex inter-relationship between different physiological systems (e.g., metabolic function, respiratory, endocrine, immunological and autonomic nervous systems, among others)^4^. Specifically, HRV refers to the variability of time intervals between normal consecutive heart beats^1^. Usually, a healthy heart shows higher beat-to-beat variability (i.e., higher HRV) compared with a less healthy heart^1,5,6^. In consequence, a reduced HRV is related to a higher morbidity (including obesity, diabetes or cardiovascular events, among others), and mortality^7,8^. Furthermore, a decrease in HRV has been related to ageing processes, especially from 40 to 60 years^9^.

HRV can be accurately recorded using non-invasive tools such as an electrocardiogram or a heart rate monitor^1,10^. These instruments have been usually employed to collect data during long-term HRV recordings (24-h), short-term HRV recordings (5–15 min) and ultra-short-term HRV recordings (< 5 min)^8,10,11^. Short-term HRV recordings at rest have gained popularity in recent years due to their high feasibility together with the capacity to obtain meaningful and accurate data. Moreover, short-term HRV recordings make easier the HRV signal acquisition under more controlled conditions (i.e., a lower number of confounder factors such as circadian rhythm, respiratory rate, body positioning…) compared with long-term HRV recordings^10,11^.

Despite recording advantages previously commented, the HRV data signal processing of short-term recordings can affect the HRV derived parameters afterwards^12^. Data processing of HRV recordings allows to partially remove both mechanical and physiological artefacts from the HRV signal recorded (e.g., ectopic beats)^1,12^. Usually, the HRV recordings are screened to manually select the 5 top-quality minutes^11,13,14^, which is defined as: (1) no presence of large R–R interval outliers, (2) equidistance between consecutive R–R intervals and (3) unimodal Gaussian R–R intervals and heart rate distribution graphics^13,14^. Thus, the HRV data signal processing of short-term recordings requires, in a greater or lesser extent, subjective decisions from the researcher, which could lead to inter- and intra-researcher differences of HRV parameters derived from short-term recordings.

To our knowledge, only two previous studies have tested either the inter- or intra-researcher reproducibility of the selected R–R interval period using the Kubios software^15,16^. Farah et al.^15^ found that the inter-researcher reproducibility was lower than the intra-researcher reproducibility in 27 healthy adolescents and concluded that the same researcher should perform the HRV data signal processing of short-term recordings^15^. In the same way, Bassi et al. reported similar results to the obtained by Farah et al.^15^ in a cohort of 44 middle-aged adults with type 2 diabetes^16^. However, little is known about how much the observers’ decisions influence HRV parameters measured with heart rate monitors (e.g., Polar) in different age-range populations including a relatively larger sample size than in the previous studies^15,16^. This is even more important since such heart rate monitors are getting more and more popular not only among researchers but also among common users. Importantly, several aspects may affect the reproducibility of HRV measurements, such as individual characteristics^17^ or technical aspects^18^. Therefore, the present work examines whether the inter- and intra-researcher reproducibility of HRV parameters derived from short-term recordings using the Kubios software has an impact on the HRV derived parameters (i.e., the influence of R–R interval selection by different researchers and by the same researcher in different moments on the quantification of HRV parameters, respectively) in three different cohorts (children with overweight/obesity [OW/OB], and healthy young and middle-aged adults).

## Method

### Participants

This study used baseline data from three different studies. Participants were 107 children with OW/OB (10.0 ± 1.1 years, 58% male) from the ActiveBrains project^19^, 132 young adults (22.2 ± 2.2 years, 33% male) from the ACTIBATE study^20^ and 73 middle-aged adults (53.6 ± 5.2 years, 48% male) from the FIT-AGEING study^21^. The inclusion/exclusion criteria have been described elsewhere^19–21^. Briefly, the inclusion criteria in common for the three studies were: (1) not being physically active; (2) not having a diagnostic of cardiovascular disease; (3) not being a smoker; (4) not being enrolled in a weight loss program; and (5) not being pregnant.

The ActiveBrains, ACTIBATE and FIT-AGEING studies were approved by the Committee for Research Involving Human Subjects at the University of Granada (References: no. 848, no. 924 for the ActiveBrains and ACTIBATE studies), Servicio Andaluz de Salud (Centro de Granada, CEI-Granada) and the Human Research Ethics Committee of the “Junta de Andalucia” [0838-N-2017] for the FIT-AGEING study. These projects were registered in the ClinicalTrials.gov (Identifiers: NCT02295072; Identifier: NCT02365129 and NCT03334357, respectively), and conducted according to the Declaration of Helsinki (revision of 2013). All participants and parents (if subjects were under 18) were informed of the purpose of the study and written informed consent was obtained.

### Anthropometric measurements

Body weight and height were measured with an electronic scale and a stadiometer, respectively (SECA instruments, model 799, Electronic Column Scale, Hamburg, Germany) prior to the HRV assessment. Body mass index (BMI) was calculated as kg/m^2^. Body weight, height and waist circumference were measured twice consecutively by the same trained researcher, and the average for each parameter was used.

### Study design

**Figure 1:**
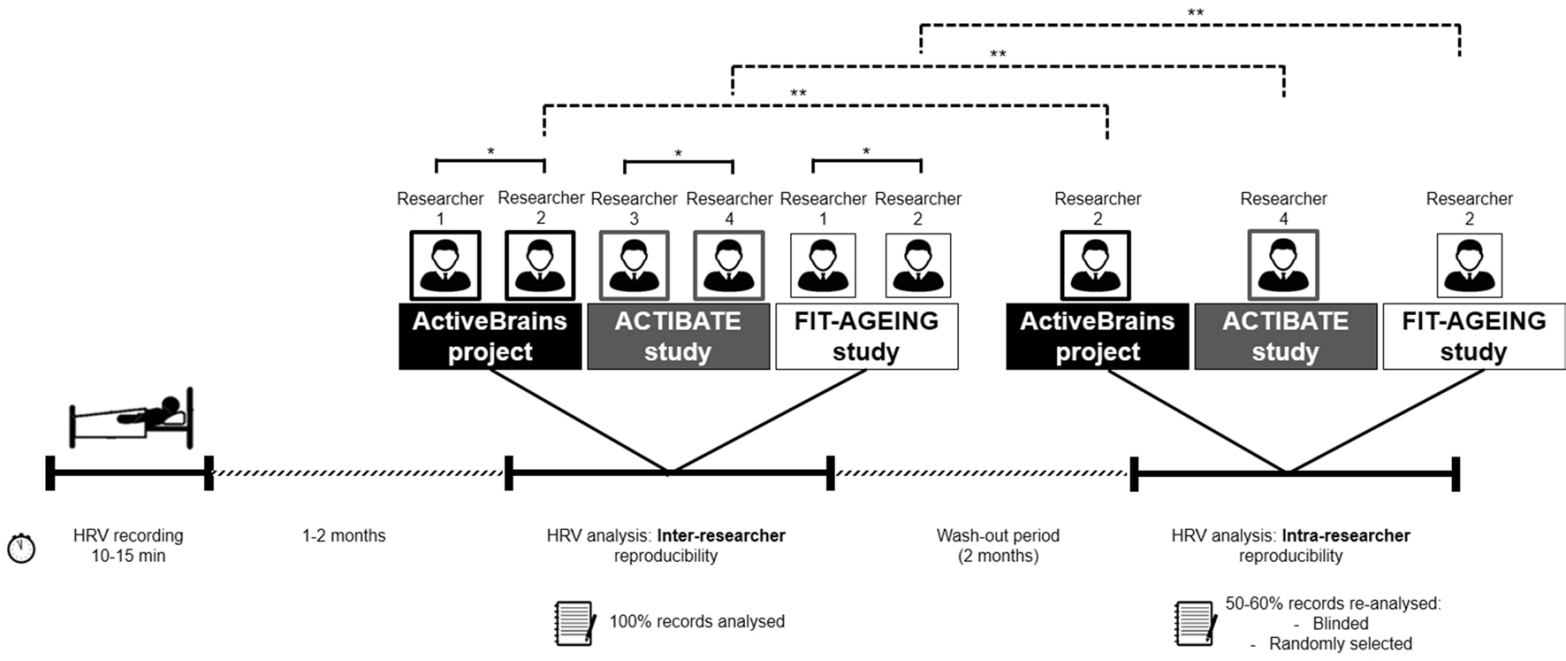
Study design. *HRV* heart rate variability; solid line plus asterisk (*) means inter-researcher reproducibility; dashed line plus asterisks (**) means intra-researcher reproducibility.

Study design is graphically presented in Fig. 1. To assess the inter-researcher reproducibility of HRV parameters derived from short-term recordings, two different trained researchers processed the data (more than 3 years of experience; background in sport sciences and exercise physiology) from the same short-term HRV recordings, (the two trained researchers could not be the same in all cohorts; see Fig. 1). To assess the intra-researcher reproducibility, the same HRV signal was processed twice by the same trained researcher 2 months apart (wash-out period). For this purpose, one of the researchers who first-processed the HRV signals was chosen to re-process the ≈ 50 to 60% of the recordings. Those recordings were randomly selected and blinded by an external researcher.

### HRV recording and data processing protocol

Participants lay in a supine position for 10–15 min while the HRV signal was recorded. HRV assessment took place in a quiet room with dim lighting, controlled ambient temperature and humidity (ACTIBATE and FIT-AGEING studies) between 8 AM and 12 PM (specifically: ActiveBrains project: 9 AM–12 PM; ACTIBATE and FIT-AGEING studies: 8 AM–9 AM). The Polar RS800CX heart rate monitor (Polar Electro Oy Inc., Kempele, Finland) was used to record the HRV signal during 10–15 min at a sampling frequency of 1,000 Hz. Participants were instructed to breathe normally, and not to fidget, talk or sleep during recordings. For HRV signal analyses we used the normal R–R intervals after excluding the extreme values with a medium filter^18^ using the Kubios software, standard version 3.2 (HRV analysis, University of Eastern Finland)^22,23^. The R–R intervals series were detrended using the smoothness prior method with lambda set at 500 and a cubic interpolation at the default rate of 4 Hz. A visual inspection was performed on the 10–15 min recorded, with each researcher manually selecting 5 min of the register (e.g., from minute 3 to minute 8) after considering that the selected 5 min met the top-quality criteria: (1) no large R–R interval outliers (i.e., a R–R interval extremely higher or lower than the whole R–R signal, based on the visual inspection of the HRV recording performed by the researcher), (2) R–R intervals equidistance (i.e., large or short distance between R–R intervals of the HRV recording, visually inspected by the researcher), and (3) Gaussians R–R intervals and heart rate distribution graphics^13,14^.

Under the Guidelines of Task Force of The European Society of Cardiology and The North American Society of Pacing and Electrophysiology recommendations^1^, we derived the most used time- and frequency-domain HRV parameters obtained from short-term recordings. In the time-domain, we computed the squared root of the mean of the sum of the squares of successive normal R–R interval differences (RMSSD) in milliseconds (ms), number of pairs of adjacent normal R–R intervals differing by more than 50 ms in the entire recording, expressed as a percentage (pNN50), and the standard deviation of all normal R–R intervals in ms (SDNN). In the frequency-domain, we performed spectral analyses using the non-parametric and parametric methods, fast Fourier transformation (FFT) and autoregressive (AR) algorithms respectively. FFT algorithm with Welch’s periodogram method (50% overlap Hanning window as pre-processing technique and calculating area under the curve with an integration) and AR with a model order of 16 as previously recommended^24^. We derived the power in the high frequency (HF: 0.15–0.4 Hz), the power in the low frequency (LF: 0.04–0.15), both in absolute units (ms^2^) and the LF/HF ratio.

The physiological interpretation of HRV parameters in time- and frequency-domain should be briefly mentioned. Usually, higher HRV values on time-domain parameters (i.e., RMSSD, pNN50 and SDNN) are considered as higher vagal tone associated with a lower cardiovascular disease risk and mortality^7^. However, the time-domain HRV parameters measure autonomic nervous system activity but cannot differentiate changes on HRV due to withdrawal of vagal tone or increased sympathetic tone and are less precise in terms of frequency delimitation^3^. On the other hand, HRV parameters in frequency-domain can quantify cyclic fluctuations of R–R intervals. The spectral analyses divide HRV into different oscillatory components (such as HF or LF). HF component reflects respiratory sinus arrhythmia, which is mainly influenced by vagal activity. LF fluctuations are due to parasympathetic and sympathetic control. The ratio LF/HF is often considered as an indicator of sympatho-vagal balance (e.g., parasympathetic withdrawal is accompanied by sympathetic activation), however, this interpretation has been recently challenged^25^.

### Statistical analysis

Descriptive data are presented as means and standard deviations (SD) for continuous variables, and frequencies and percentages for categorical variables. Normal distribution of the included variables was tested using the Kolmogorov–Smirnov test, and visual inspection of histograms was also performed. HRV parameters derived from short-term recordings did not exhibit normal distributions, however, since sensibility analyses did not show differences after normalizing the variables, data were analyzed and presented as non-normalized values. Intraclass correlation coefficient (ICC) confidence interval (95% IC), inter- and intra-coefficient of variation (CV), Bland–Altman plots (mean difference [bias] and limits of agreement)^26^ and the root mean square error (RMSE) were used to study the inter- and intra-researcher reproducibility for HRV parameters derived from short-term recordings. Heteroscedasticity, or whether the variability (error) increases or decreases as the magnitude of the measure increase, was calculated using linear regressions between the mean values and the absolute values of HRV parameters differences. Of note, all the analyses were performed separately because we observed an interaction *age* × *study population* effect, influencing most of the HRV parameters (P < 0.05). All the analyses were conducted using the Statistical Package for Social Sciences (SPSS, v. 21.0, IBM SPSS Statistics, IBM Corporation).

## Results

Table 1 shows participants’ characteristics. Table 2 shows the inter-researcher reproducibility of HRV parameters derived from short-term recordings in time- and frequency-domains. The ICCs of HRV parameters in time-domain ranged from 0.822 to 0.989, while for the frequency-domain ranged from 0.703 to 0.985. Overall, the CVs for HRV parameters in the time-domain were lower than 5%, except for the pNN50 in the young and middle-aged adults’ cohorts, in which it was lower than 6 and 9%, respectively. CVs for HRV parameters in frequency-domain were lower than 16% for children, 17% for young adults and 21% for middle-aged adults in all cases. RMSE of the HRV parameters in time-domain ranged from 2.66 to 9.17, while for the frequency-domain ranged from 154.19 to 839.51 (HF and LF respectively) and from 0.32 to 2.38 (ratio LF/HF).

**Table 1:** Participants’ descriptive characteristics.

	Children with OW/OB (n = 107)	Young adults (n = 132)	Middle-aged adults (n = 73)
Age (years)	10.03 ± 1.13	22.22 ± 2.20	53.62 ± 5.18
Sex (n; % male)	62; 57.9	43; 32.6	35; 47.9
Body weight (kg)	56.05 ± 11.06	71.39 ± 17.14	75.63 ± 15.06
Height (m)	144.13 ± 8.36	168.29 ± 8.65	167.86 ± 9.88
Body mass index (kg/m^2^)	26.77 ± 3.62	25.04 ± 4.77	26.67 ± 3.76
Waist circumference (cm)	90.95 ± 9.75	80.34 ± 17.59	94.99 ± 11.79

Data are presented as mean and standard deviation, otherwise stated.

*OW/OB* overweight/obesity.

**Table 2:** Inter-researcher reproducibility of HRV parameters derived from short-term recordings.

	Researcher 1 Mean ± stand. deviation	Researcher 2 Mean ± stand. deviation	Mean difference ± stand. deviation	95% limits of agreement	ICC (95% CI)	CV (%)	RMSE
**Children with overweight/obesity (n = 107)**							
RMSSD (ms)	61.66 ± 33.22	59.76 ± 30.66	1.71 ± 8.20	− 14.38; 17.81	0.966 (0.950;0.977)	3.8	9.17
pNN50 (%)	30.45 ± 20.37	29.98 ± 19.75	0.31 ± 2.91	− 5.40; 6.02	0.989 (0.985;0.993)	3.9	3.13
SDNN (ms)	59.04 ± 25.98	57.74 ± 24.53	1.29 ± 6.48	− 11.41; 13.99	0.966 (0.951;0.977)	3.3	8.47
FFT_HF (ms^2^)	1898.07 ± 1937.11	1767.70 ± 1744.89	126.79 ± 736.13	− 1,316.02; 1569.60	0.919 (0.883;0.994)	15.9	752.02
AR_HF (ms^2^)	1855.29 ± 1852.42	1756.60 ± 1698.83	92.62 ± 641.94	− 1,165.58; 1,350.81	0.934 (0.905;0.955)	7.8	651.39
FFT_LF (ms^2^)	1688.39 ± 1599.80	1674.03 ± 1557.16	34.20 ± 456.80	− 861.13; 929.53	0.958 (0.939;0.971)	15.3	582.68
AR_LF (ms^2^)	1538.49 ± 1,348.11	1566.66 ± 1,371.32	− 22.02 ± 331.67	− 672.10; 628.05	0.971 (0.957;0.980)	8.4	392.76
FFT_LF/HF	1.27 ± 0.94	1.28 ± 0.82	0.00 ± 0.53	− 1.03; 1.03	0.823 (0.751;0.876)	14.9	0.58
AR_LF/HF	1.14 ± 0.71	1.18 ± 0.69	− 0.04 ± 0.32	− 0.66; 0.59	0.895 (0.850;0.927)	10.5	0.37
**Young adults (n = 132)**							
RMSSD (ms)	58.75 ± 32.16	60.20 ± 31.49	− 1.45 ± 4.71	− 10.68; 7.78	0.988 (0.982;0.992)	3.3	4.91
pNN50 (%)	33.24 ± 22.25	34.07 ± 21.90	− 0.82 ± 3.69	− 8.06; 6.41	0.985 (0.979;0.990)	6.0	4.55
SDNN (ms)	52.20 ± 23.86	53.89 ± 23.95	− 1.69 ± 4.24	− 10.00; 6.62	0.982 (0.969;0.989)	3.6	3.77
FFT_HF (ms^2^)	1594.27 ± 1789.73	1,700.78 ± 1814.40	− 106.51 ± 420.69	− 931.07; 718.05	0.971 (0.958;0.980)	12.5	432.42
AR_HF (ms^2^)	1626.19 ± 1777.48	1675.50 ± 1717.53	− 49.31 ± 295.96	− 629.39; 530.76	0.985 (0.979;0.990)	8.0	298.93
FFT_LF (ms^2^)	1,235.20 ± 1,307.97	1,350.06 ± 1,493.56	− 144.86 ± 874.79	− 1751.04; 1521.32	0.822 (0.757;0.870)	17.2	839.51
AR_LF (ms^2^)	1,237.36 ± 1,330.89	1,349.15 ± 1,464.88	− 111.79 ± 410.93	− 917.22; 693.63	0.954 (0.933;0.968)	10.8	424.36
FFT_LF/HF	1.20 ± 1.21	1.15 ± 1.02	0.05 ± 0.64	− 1.21; 1.31	0.834 (0.773;0.879)	16.7	0.64
AR_LF/HF	1.14 ± 0.96	1.12 ± 0.87	0.02 ± 0.32	− 0.61; 0.65	0.939 (0.916;0.957)	10.5	0.32
**Middle-aged adults (n = 73)**							
RMSSD (ms)	31.33 ± 19.54	32.43 ± 19.54	− 0.94 ± 4.74	− 10.23; 8.34	0.970 (0.952;0.981)	4.7	4.77
pNN50 (%)	12.80 ± 16.23	13.25 ± 16.22	− 0.30 ± 2.67	− 5.52; 4.93	0.987 (0.979;0.992)	8.9	2.66
SDNN (ms)	30.18 ± 13.95	31.36 ± 13.94	− 1.05 ± 5.22	− 11.28; 9.19	0.928 (0.888;0.955)	5.1	5.29
FFT_HF (ms^2^)	462.42 ± 607.67	492.49 ± 617.23	− 25.57 ± 200.10	− 417.75; 366.62	0.947 (0.916;0.966)	16.0	200.34
AR_HF (ms^2^)	473.05 ± 624.58	488.12 ± 585.05	− 10.66 ± 154.91	− 314.28; 292.96	0.968 (0.949;0.980)	9.7	154.19
FFT_LF (ms^2^)	469.06 ± 435.09	505.92 ± 463.28	− 25.86 ± 246.89	− 509.76; 458.05	0.850 (0.771;0.903)	21.1	246.53
AR_LF (ms^2^)	466.17 ± 442.23	505.27 ± 459.28	− 29.05 ± 200.98	− 422.97; 364.87	0.900 (0.846;0.936)	11.7	201.68
FFT_LF/HF	2.22 ± 3.29	2.03 ± 2.88	0.19 ± 2.39	− 4.50; 4.89	0.703 (0.564;0.803)	18.3	2.38
AR_LF/HF	1.98 ± 2.19	2.08 ± 2.79	− 0.13 ± 0.97	− 2.04; 1.78	0.925 (0.882;0.952)	12.8	0.98

*HRV* heart rate variability, *RMSSD* the square root of the mean of the sum of the squares of differences between adjacent R–R intervals, *pNN50* percentage (%) of the total pairs of R–R intervals that differ by more than 50 ms, *SDNN* standard deviation of all normal R–R intervals, *FFT* fast Fourier Transformation algorithm, *HF* high frequency (0.15–0.4 Hz) index of parasympathetic activity, *LF* low frequency (0.04–0.15 Hz) mix index of sympathetic and parasympathetic activity, *AR* autoregressive algorithm, *ms* milliseconds, *CV* coefficient of variation, *ICC* intraclass correlation coefficient, *RMSE* root mean square error.

Table 3 shows the intra-researcher reproducibility of HRV parameters derived from short-term recordings. The ICCs of HRV parameters in time-domain ranged from 0.972 to 0.998, while for the frequency-domain ranged from 0.950 to 0.996. The CVs of HRV parameters in time-domain were lower than 4% in most HRV parameters, except for pNN50 in the middle-aged adult’s cohort (11%). CVs of the HRV parameters in frequency-domain were lower than 4% for children, 11% for young adults and 14% for middle-aged adults in all cases. On average, the intra-researcher differences were 31%, 62%, and 80% smaller than the inter-researcher differences based on CVs in children with OW/OB, young and middle-aged adults, respectively (data not shown). RMSE of the HRV parameters in time-domain ranged from 1.09 to 3.21, while for the frequency-domain ranged from 113.05 to 402.13 (HF and LF respectively) and from 0.22 to 0.93 (ratio LF/HF).

**Table 3:** Intra-researcher reproducibility of HRV parameters derived from short-term recordings.

	1st processing Mean ± stand. deviation	2nd processing Mean ± stand. deviation	Mean difference ± stand. deviation	95% Limits of agreement	ICC (95% CI)	CV (%)	RMSE
**Children with overweight/obesity (n = 99)**							
RMSSD (ms)	59.76 ± 30.66	59.86 ± 30.93	0.32 ± 2.47	− 4.52; 5.15	0.997 (0.995;0.998)	1.1	2.48
pNN50 (%)	29.98 ± 19.75	30.18 ± 20.07	0.29 ± 2.35	− 4.32; 4.89	0.998 (0.998;0.999)	1.2	1.09
SDNN (ms)	57.74 ± 24.53	57.56 ± 24.28	0.09 ± 1.11	− 2.09; 2.28	0.995 (0.993;0.997)	1.0	2.35
FFT_HF (ms^2^)	1767.70 ± 1744.89	1813.02 ± 1813.98	3.58 ± 158.87	− 307.80; 314.97	0.996 (0.994;0.997)	3.0	157.31
AR_HF (ms^2^)	1756.60 ± 1698.83	1772.00 ± 1728.91	19.41 ± 275.68	− 520.92; 559.75	0.987 (0.981;0.992)	2.4	274.97
FFT_LF (ms^2^)	1674.03 ± 1557.16	1674.92 ± 1,590.28	− 24.25 ± 266.75	− 547.08; 498.58	0.985 (0.978;0.990)	4.3	266.50
AR_LF (ms^2^)	1566.66 ± 1,371.32	1,520.93 ± 1,333.45	30.66 ± 247.76	− 454.93; 516.26	0.983 (0.975;0.988)	3.7	248.40
FFT_LF/HF	1.28 ± 0.82	1.25 ± 0.80	0.02 ± 0.26	− 0.48; 0.52	0.952 (0.930;0.968)	3.9	0.24
AR_LF/HF	1.18 ± 0.69	1.15 ± 0.68	0.03 ± 0.22	− 0.40; 0.45	0.950 (0.926;0.966)	3.9	0.22
**Young adults (n = 73)**							
RMSSD (ms)	60.20 ± 31.49	59.27 ± 29.23	− 0.46 ± 2.77	− 4.96; 5.88	0.995 (0.993;0.997)	1.8	2.90
pNN50 (%)	34.07 ± 21.90	33.88 ± 20.96	0.44 ± 2.22	− 3.90; 4.79	0.994 (0.991;0.996)	3.0	2.25
SDNN (ms)	53.89 ± 23.95	52.71 ± 22.01	0.50 ± 3.11	− 5.94; 6.59	0.990 (0.984;0.994)	2.1	3.13
FFT_HF (ms^2^)	1,700.78 ± 1814.40	1584.71 ± 1948.83	− 8.49 ± 327.44	− 650.27; 633.29	0.986 (0.977;0.991)	8.5	325.24
AR_HF (ms^2^)	1675.50 ± 1717.53	1592.50 ± 1737.96	− 38.89 ± 156.37	− 345.38; 267.59	0.996 (0.993;0.997)	4.6	160.06
FFT_LF (ms^2^)	1,350.06 ± 1,493.56	1,251.76 ± 1,394.01	52.20 ± 384.82	− 701.05; 805.46	0.965 (0.945;0.978)	10.9	402.13
AR_LF (ms^2^)	1,349.15 ± 1,464.88	1,278.26 ± 1,376.76	22.24 ± 262.34	− 491.95; 536.42	0.982 (0.971;0.989)	7.7	272.81
FFT_LF/HF	1.15 ± 1.02	1.25 ± 1.12	− 0.03 ± 0.38	− 0.77; 0.70	0.943 (0.910;0.964)	10.5	0.38
AR_LF/HF	1.12 ± 0.87	1.15 ± 0.94	0.01 ± 0.28	− 0.53; 0.56	0.955 (0.928;0.971)	7.9	0.28
**Middle-aged adults (n = 39)**							
RMSSD (ms)	32.43 ± 19.54	32.04 ± 18.28	− 0.87 ± 2.96	− 6.67; 4.92	0.985 (0.972;0.992)	3.8	3.06
pNN50 (%)	13.25 ± 16.22	12.72 ± 14.97	− 0.82 ± 3.13	− 6.96; 5.33	0.976 (0.955;0.987)	10.9	3.20
SDNN (ms)	31.36 ± 13.94	31.77 ± 13.59	0.04 ± 3.25	− 6.32; 6.41	0.972 (0.948;0.985)	4.3	3.21
FFT_HF (ms^2^)	492.49 ± 617.23	479.04 ± 598.83	− 21.53 ± 112.43	− 241.90; 198.84	0.982 (0.966;0.990)	12.2	113.05
AR_HF (ms^2^)	488.12 ± 585.05	503.54 ± 649.73	− 47.62 ± 177.95	− 396.40; 301.17	0.976 (0.953;0.987)	7.4	181.94
FFT_LF (ms^2^)	505.92 ± 463.28	571.89 ± 516.77	− 9.56 ± 166.45	− 335.80; 316.69	0.951 (0.909;0.974)	13.7	164.58
AR_LF (ms^2^)	505.27 ± 459.28	541.69 ± 471.18	33.80 ± 127.73	− 216.55; 284.16	0.966 (0.936;0.982)	8.8	130.54
FFT_LF/HF	2.03 ± 2.88	2.39 ± 3.58	− 0.07 ± 0.35	− 0.76; 0.62	0.995 (0.990;0.997)	10.8	0.36
AR_LF/HF	2.08 ± 2.79	2.23 ± 3.17	0.24 ± 0.91	− 1.54; 2.03	0.957 (0.919;0.977)	10.4	0.93

*HRV* heart rate variability, *RMSSD* the square root of the mean of the sum of the squares of differences between adjacent R–R intervals, *pNN50* percentage (%) of the total pairs of R–R intervals that differ by more than 50 ms, *SDNN* standard deviation of all normal R–R intervals, *FFT* fast Fourier Transformation algorithm, *HF* high frequency (0.15–0.4 Hz) index of parasympathetic activity, *LF* low frequency (0.04–0.15 Hz) mix index of sympathetic and parasympathetic activity, *AR* autoregressive algorithm, *ms* milliseconds, *CV* coefficient of variation, *ICC* intraclass correlation coefficient, *RMSE* root mean square error.

**Figure 2:**
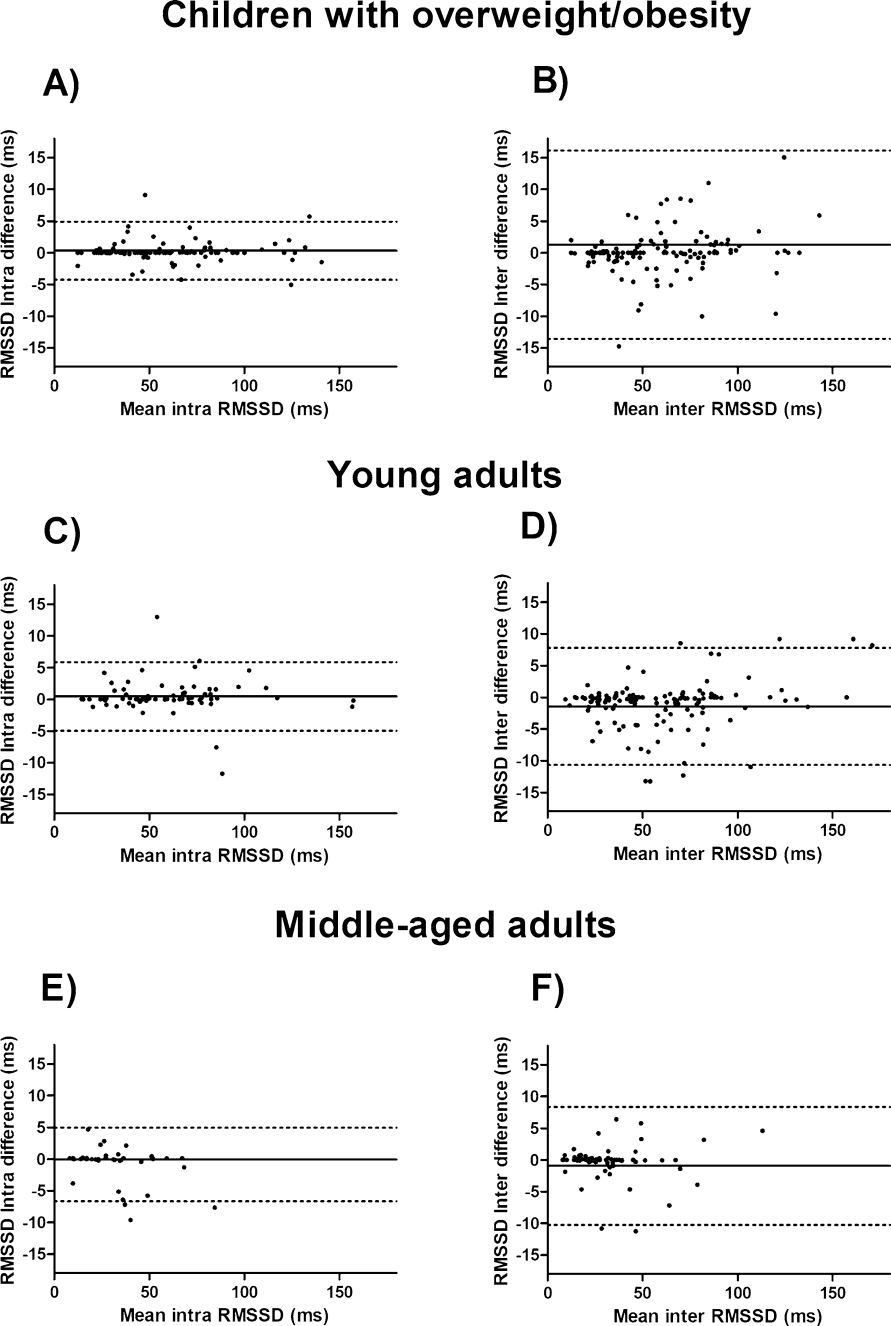
Bland–Altman plots for the squared root of the mean of the sum of the squares of successive normal R–R interval differences (RMSSD) in ms in different cohorts: (**A**,**B**) are for children with OW/OB, (**C**,**D**) are for young adults and, (**E**,**F**) are for middle-aged adults cohorts respectively.

**Figure 3:**
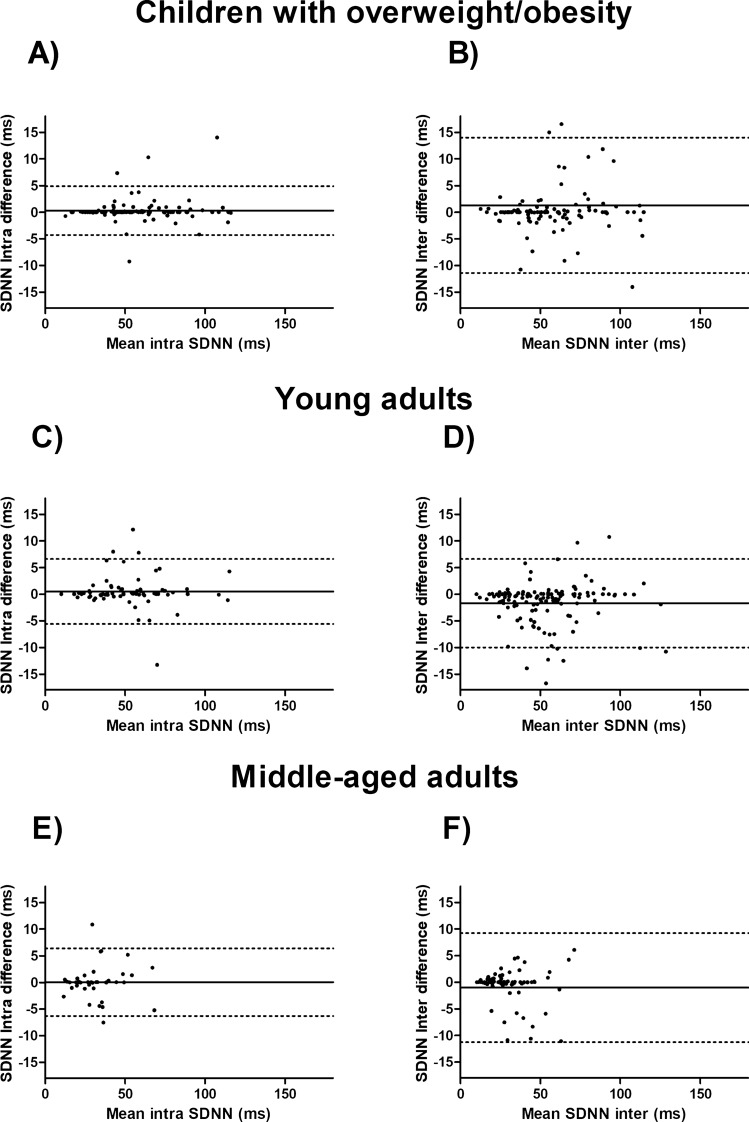
Bland–Altman plots for standard deviation of all normal R–R intervals (SDNN) in ms in different populations: (**A**,**B**) are for children with OW/OB, (**C**,**D**) for young adults and, (**E**,**F**) for middle-aged adult cohorts respectively.

Figures 2 and 3 shows Bland–Altman plots for RMSSD and SDNN respectively. Mean inter- differences and limits of agreement (95%) for RMSSD were 1.71; (− 14.38; 17.81) for children with OW/OB, − 1.45; (− 10.68; 7.78) for young adults and, − 0.94; (− 10.23; 8.34) for middle-aged adults. Whereas mean intra-differences and limits of agreement (95%) for RMSSD were 0.32; (− 4.52; 5.15) for children with OW/OB, − 0.46; (− 4.96; 5.88) for young adults and, − 0.87; (− 6.67; 4.92) for middle-aged adults. Moreover, the inter-researcher difference was higher in children with OW/OB, 1.71; (− 14.38; 17.81), compared with young and middle-aged adults, − 1.45; (− 10.68; 7.78) and − 0.94; (− 10.23; 8.34), respectively. We did not detect heteroscedasticity on inter- and intra-reproducibility analyses (all p > 0.05, Figs. 2 and 3) except for inter-reproducibility analyses for RMSSD in children with OW/OB (β = 0.304, p < 0.001; Fig. 2B). These patterns in Bland–Altman plots were similar for SDNN (see Fig. 3). Bland–Altman plots for the pNN50 and for HRV parameters derived from the frequency-domain are presented as Supplementary Material (see Figures S1–S7).

## Discussion

The main findings of our research are: (1) this study provides the quantification of the inter-researcher and intra-researcher differences (i.e., the influence of R–R interval selection by different researchers and by the same researcher in different moments on the quantification of HRV parameters, respectively) in three different human cohorts. Although the inter- and intra-researcher differences were not very large, the inter-researcher reproducibility could elicit clinical relevant differences for some HRV parameters (e.g., some differences on SDNN values ≥ 10 ms^27^ based on limits of agreement in Bland Altman plots); (2) overall, HRV parameters in frequency-domain derived from short-term recordings presented lower reproducibility compared with those in time-domain in both the inter- and intra-researcher processing. Therefore, the data obtained from our three cohorts support the idea that HRV signal processing should be performed by the same trained researcher to obtain consistent and reproducible HRV parameters derived from short-term recordings in time- and frequency-domain. Alternatively, automatic algorithms for an ECG segment selection should be developed to overcome the reproducibility problem.

To our knowledge, only three previous studies focused on inter- and intra-researcher reproducibility for HRV derived parameters from long-term recordings^28–30^, whilst only two previous study focused on short-term recordings^15,16^. We observed similar findings like those reported by Farah et al.^15^ and Bassi et al.^16^ in a sample of 27 healthy adolescents and 44 middle-aged adults with type 2 diabetes, respectively. Of note, Farah et al.^15^ registered the HRV signal using the same heart rate monitor (Polar RS800CX) and performed the same statistical tests to study inter-and intra-researcher reproducibility of HRV parameters derived from short-term recordings.

In the study conducted by Farah et al.^15^ carried out in adolescents, ICCs ranged from 0.915 to 0.996 and 0.990 to 0.993 for inter- and intra-researcher reproducibility, respectively, while for children with OW/OB our ICCs ranged from 0.823 to 0.989 and 0.950 to 0.998 for inter- and intra-researcher reproducibility, respectively. Likewise, CVs reported by Farah et al.^15^ were lower than 20% and 7% for inter-and intra-researcher reproducibility, respectively. We found similar CVs in children, i.e., lower than 15.9% and 4.3% for inter-and intra-researcher reproducibility, respectively. The marginal differences observed across studies could be partially explained by the different biological characteristics’ between them (e.g., children with OW/OB vs. adolescents) and/or by some methodological inconsistencies in HRV data collection (i.e., standard operation procedure) and/or HRV data signal processing.

In regards to young adults, in our study, ICCs ranged from 0.822 to 0.985 and 0.990 to 0.996 for inter- and intra-researcher reproducibility respectively. Likewise, the CVs were lower than 16.7% and 10.9% for inter-and intra-researcher reproducibility. On the other hand, respect to middle-aged adults, ICCs ranged from 0.850 to 0.987 (with the exception of the FFT_LF/HF parameter; ICCs 0.703) and 0.951 to 0.995 for inter- and intra-researcher reproducibility, respectively. Similarly to our findings in healthy middle-aged adults, Bassi et al.^16^ reported ICC ranging from 0.730 to 0.970 and 0.910 to 0.990 for inter- and intra-researcher reproducibility in middle-aged adults with type 2 diabetes. The little variation observed of ICCs between both studies in middle-aged adults could be partially explained by the health status of the study populations (i.e., healthy vs. type 2 diabetes) and by some methodological considerations, e.g., different model of the heart rate monitors used (Polar RS800CX vs. Polar S810i model).

The inter-researcher reproducibility of HRV parameters derived from short-term recordings was lower than intra-researcher reproducibility in our study, which could be explained by one issue related to the HRV data signal processing. Based on our previous experience in the HRV data signal processing from short-term recordings using the Kubios software, we found that, in HRV recordings presenting a high HRV (e.g., children), a Gaussian distribution of R–R intervals and HR distribution graphs is difficult to be found. Moreover, this higher HRV could complicate the decision-taking from researchers that guarantee the selection of the same best-quality 5 min, and this fact could produce the mentioned differences between HRV parameters derived from short-term recordings.

In regards to intra-researcher reproducibility, we reported CVs lower than 4.3%, 10.9% and 13.7% for children with OW/OB, healthy young and middle-aged adults respectively, in some of the HRV parameters derived from short-term recordings. Thus, in general the intra-researcher reproducibility was higher (i.e., lower CVs) in children with OW/OB in comparison with young and middle-aged adults. We expected that individuals with a higher HRV (e.g., children and young adults) presented worse intra-researcher reproducibility, in other words a higher variability between test and retest, than in individuals with lower HRV values (e.g., middle-aged or elderly adults). Hence, a higher HRV might complicates the selection of the same best-quality 5 min across researchers. In fact, in our study, the differences observed across cohorts in the intra-researcher reproducibility could be explained, in a greater or lesser extent, by methodological aspects, e.g., a different researcher performs the intra-researcher HRV signal data processing in each cohort (Fig. 1). However all of them were trained in the HRV data signal processing using the Kubios software (more than 3 years of experience; background in sport sciences and exercise physiology).

On the other hand, the Bland–Altman plots for time-domain HRV parameters derived from short-term recordings, i.e., RMSSD and SDNN (Figs. 2, 3), showed wider limits of agreement in the inter-researcher HRV signal data processing in comparison with the intra-researcher. These results are in concordance with the aforementioned statement, a higher HRV can complicate the selection of the same fragment best-quality 5 min’ period between different researchers and, this could negatively affect the reproducibility of HRV parameters derived from short-term recordings. We should consider that the ICC and CVs previously mentioned and discussed against the Farah et al.^15^ study in adolescents, include HRV parameters derived from short-term recordings in time- and frequency-domain. On the other hand, for Bland–Altman plots, we only took into account in the main text the HRV parameters derived from short-term recordings in time-domain more frequently used such as RMSDD and SDNN^1,10^ (see Figs. 2, 3).

Clinicians and researchers care about whether the magnitude of the differences elicited by the inter- and intra-researcher HRV data signal processing are clinically relevant. However, it is difficult to determine due to several methodological differences between studies related to the HRV data collection and HRV data signal processing (e.g., instrument employed to record the HRV signal, length of the recordings, HRV parameters derived reported, etc.)^31^.

Despite those methodological issues, it is relevant to take into consideration the absolute values of inter-and intra-researcher differences (see Figs. 2, 3 for a graphical visualization). In this regard, a mean increase of 6–10 ms on RMSSD has been reported after different kinds of interventions in children with OB, and in healthy and unhealthy adults^32–34^. For example, for RMSSD inter-and intra-researcher HRV signal data processing, we reported a mean difference of − 1.71 ms and 0.32 ms in children with OW/OB, respectively. Thus, based on the previous studies^32–34^, we can assume that these differences are not clinically relevant. However, limits of agreement describe inter-researcher differences up to 17.31 ms, i.e., twice the change reported in the previous mentioned interventions^32–34^, reaching intra-researcher differences a maximum of 5.15 ms. These findings question whether the nature of the change reported in the intervention could be produced by the intervention itself in a same extent that it could be produced by unreliable HRV data signal processing. To define the trained level or experience of the researchers and how many researchers were in charge of the HRV data signal processing in each time point of an intervention is a matter of main interest that should be reported in all the intervention studies.

Otherwise, Bilchick et al.^27^ reported that each increase of 10 ms in SDNN supposed a reduction of 20% of the risk of mortality in adults with ischemic cardiomyopathy (24 h- holter recordings). Importantly, the mean values of the inter- and intra-researcher differences for SDNN in our study were 1.05–1.69 ms and 0.04–0.50 ms, respectively. However, the limits of agreement showed that some inter-researcher differences could reach 11.28 ms in young adults, where intra-researcher analysis can reach a maximum difference of 6.59 ms. Thus, attending to our findings, inter-researcher differences could be clinically relevant whereas intra-researcher differences could not.

We encourage researchers to report the HRV signal data processing in detail (i.e., level of artefact correction, smoothness prior alpha value, interpolation value) including if the same or a different researcher carried out the HRV processing, as well as their experience (i.e., trained or not). Further studies are needed to test the impact of other decisions related to the HRV signal data processing such as different levels of artefact correction or experience status of the researchers on the quantification of HRV derived parameters.

Some limitations need to be mentioned: (1) the sample from ActiveBrains project were children with OW/OB, so we cannot extrapolate our findings to thinner children; (2) the intra-researcher HRV signal data processing was not performed by the same researcher in the three different cohorts, however, all the researchers in our study were trained in HRV signal data processing using the Kubios software; (3) the experimental conditions during the data acquisition were not identical in the different studies as different standard operation procedures were used (e.g., the hour and dates of the HRV assessments, etc.). On the other hand, the strengths of our study were: (1) relatively larger sample size compared to previous studies that analyzed inter- and intra-researcher reproducibility of HRV parameters derived from short-term and long-term recordings; (2) inter- and intra-researcher reproducibility of HRV parameters derived from short-term recordings were tested in three different cohorts and performing an identical methodology of HRV signal data processing and following the current guidelines for HRV assessment^1,35^.

## Conclusion

In conclusion, our study provides the quantification of the inter-researcher and intra-researcher differences (i.e., the influence of R–R interval selection by different researchers and by the same researcher in different moments on the quantification of HRV parameters, respectively) in three different human cohorts. Although the inter- and intra-researcher differences were not very large, the inter-researcher reproducibility could elicit some clinically relevant differences for some HRV parameters in time-domain. Based on our findings, we recommend that the HRV signal data processing should be performed by the same trained researcher to obtain more reproducible and consistent HRV parameters derived from short-term recordings. Alternatively, automatic algorithms for an ECG segment selection should be developed to overcome the reproducibility problem. These findings need to be considered with caution due to the three different cohorts selected from three studies with different objectives and inclusions criteria.

## Supplementary information

Supplementary information

## Abbreviations

CV: Coefficient of variation
FFT: Fast Fourier transformation
HRV: Heart rate variability
ICC: Intraclass correlation coefficient
ms: Miliseconds
OW/OB: Overweight/obesity
pNN50: Number of pairs of adjacent normal R-R intervals differing by more than 50 ms
RMSSD: Squared root of the mean of the sum of the squares of successive normal R–R interval differences
SDNN: Standard deviation of all normal RR intervals
HF: High frequency
LF: Low frequency
