# Supplementary material for: Inter- and intra-researcher reproducibility of heart rate variability parameters in three human cohorts
Source: Sci Rep. 2020 Jul 9;10:11399. doi: 10.1038/s41598-020-68197-7 (PMC7347623; doi:10.1038/s41598-020-68197-7)
Supplement: Supplementary file 1 — Supplementary information [file 41598_2020_68197_MOESM1_ESM.pdf]

Inter- and intra-researcher reproducibility of heart rate variability parameters in three human cohorts

Abel Plaza-Florido [1*†,], Juan M.A. Alcantara [1†], Jairo H. Migueles [1], Francisco J. Amaro-Gahete[1,2], Francisco M. Acosta [1], Jose Mora-Gonzalez [1], Jerzy Sacha [3,4], Francisco B. Ortega [1]

[1] PROFITH "PROmoting FITness and Health Through Physical Activity" Research Group, Sport and Health University Research Institute (iMUDS), Department of Physical and Sports Education, Faculty of Sport Sciences, University of Granada, Granada, Spain´

[2] EFFECTS-262 Research Group, Department of Physiology, School of Medicine, University of Granada, 18071 Granada, Spain

[3] Faculty of Physical Education and Physiotherapy, Opole University of Technology, Opole, Poland

[4] Department of Cardiology, University Hospital in Opole, University of Opole, Opole, Poland

† In equal contribution

***Correspondence:** Abel Plaza-Florido. E-mail: abeladrian@ugr.es
Department of Physical and Sports Education, Faculty of Sports Science, University of Granada, Granada, Spain. *Carretera de Alfacar, s/n CP: 18071 Granada.* Tlf.: *(+34) 958 24 43 53*

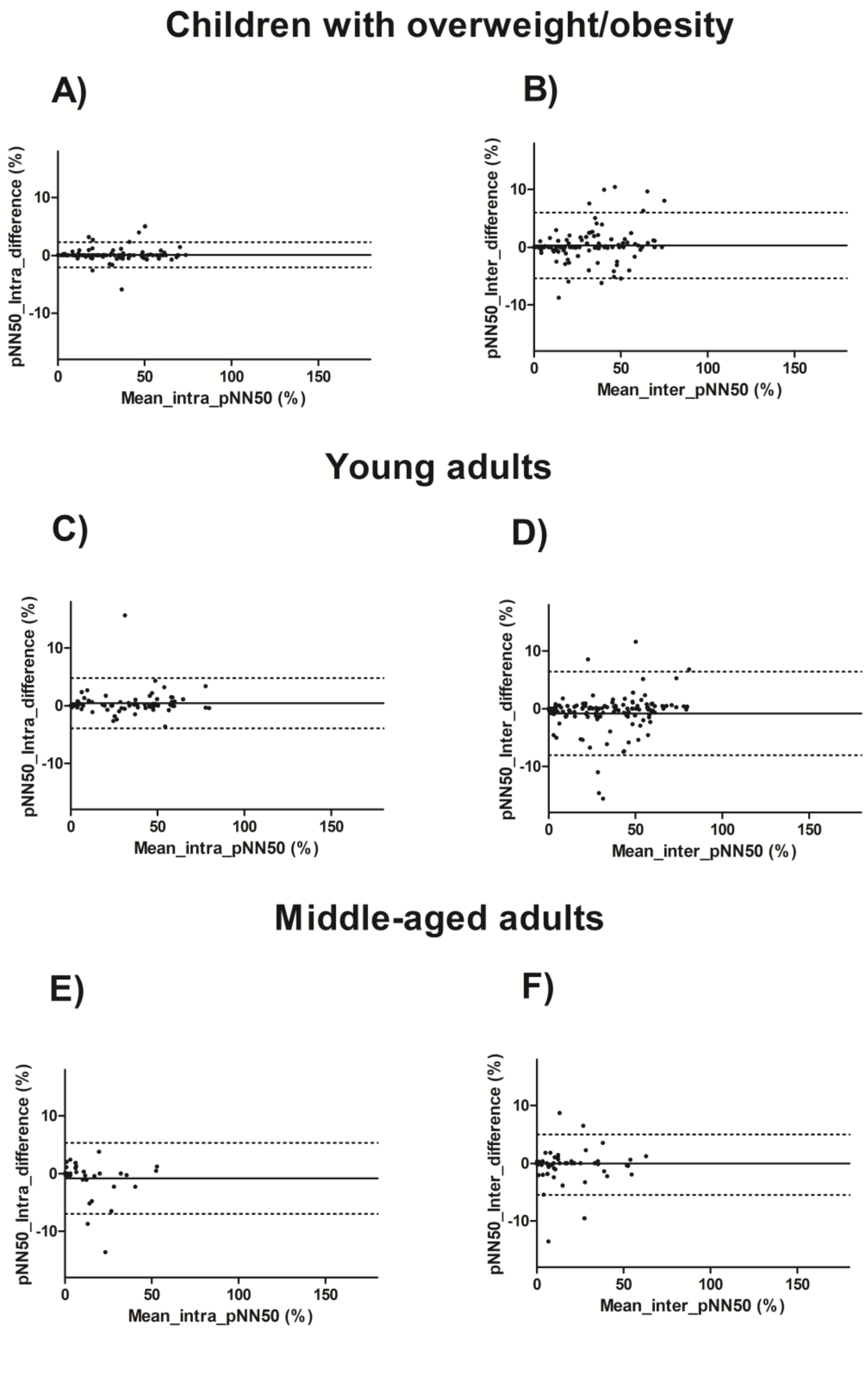

Figure S1. Bland-Altman plots for number of pairs of adjacent normal R-R intervals differing by more than 50 ms in the entire recording, expressed as a percentage (pNN50) in different populations: panels A and B) are for children with OW/OB, panels C and D) for young adults and, panels E and F) for middle-aged adult cohorts respectively.

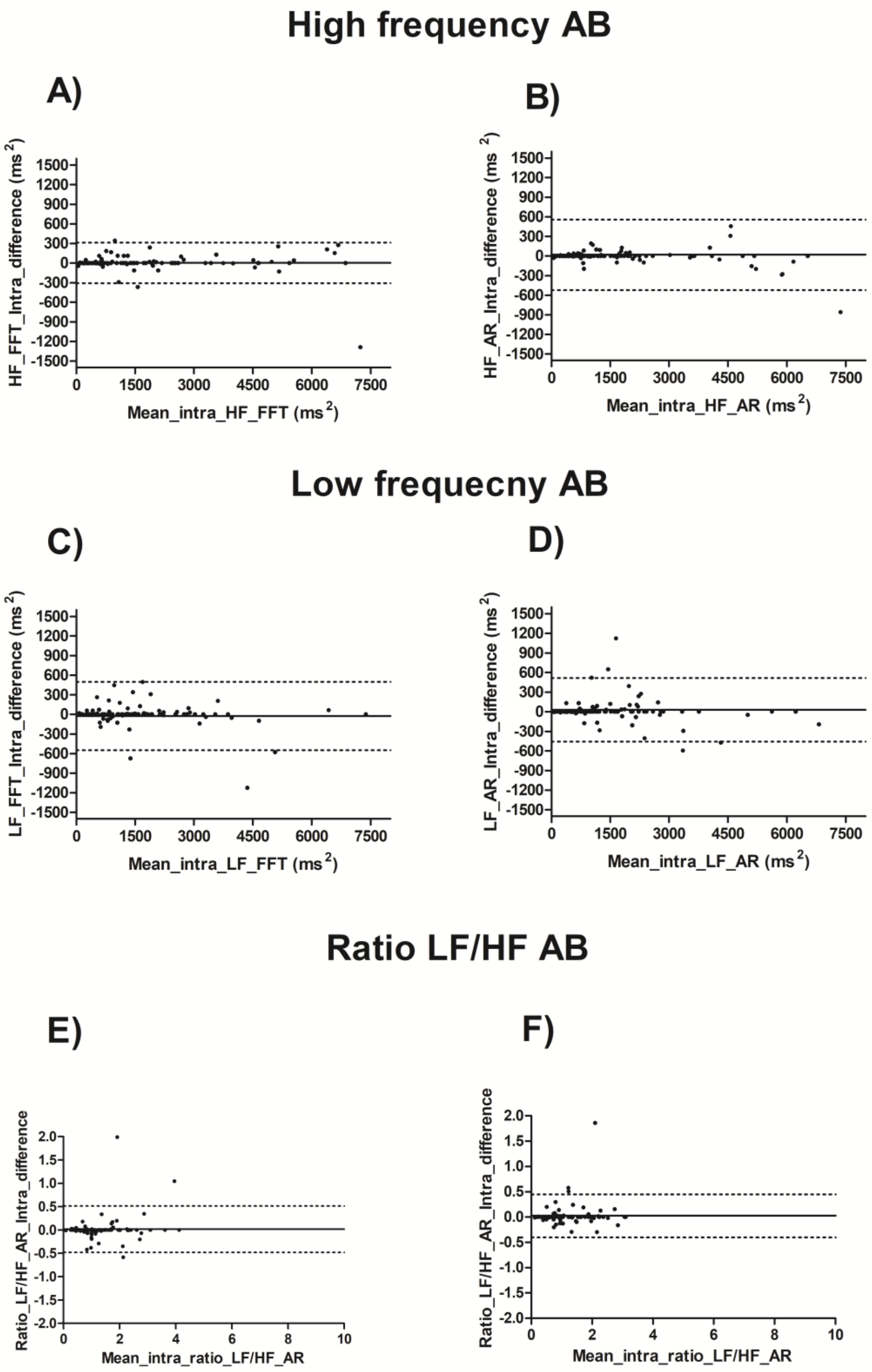

Figure S2. Bland-Altman plots for frequency-domain HRV parameters (intra-reliability) in children with OW/OB, ActiveBrains project (AB): panels A and B) are for high-frequency, panels C and D) low-frequency and, panels E and F) for ratio LF/HF with FFT and AR algorithms, respectively.

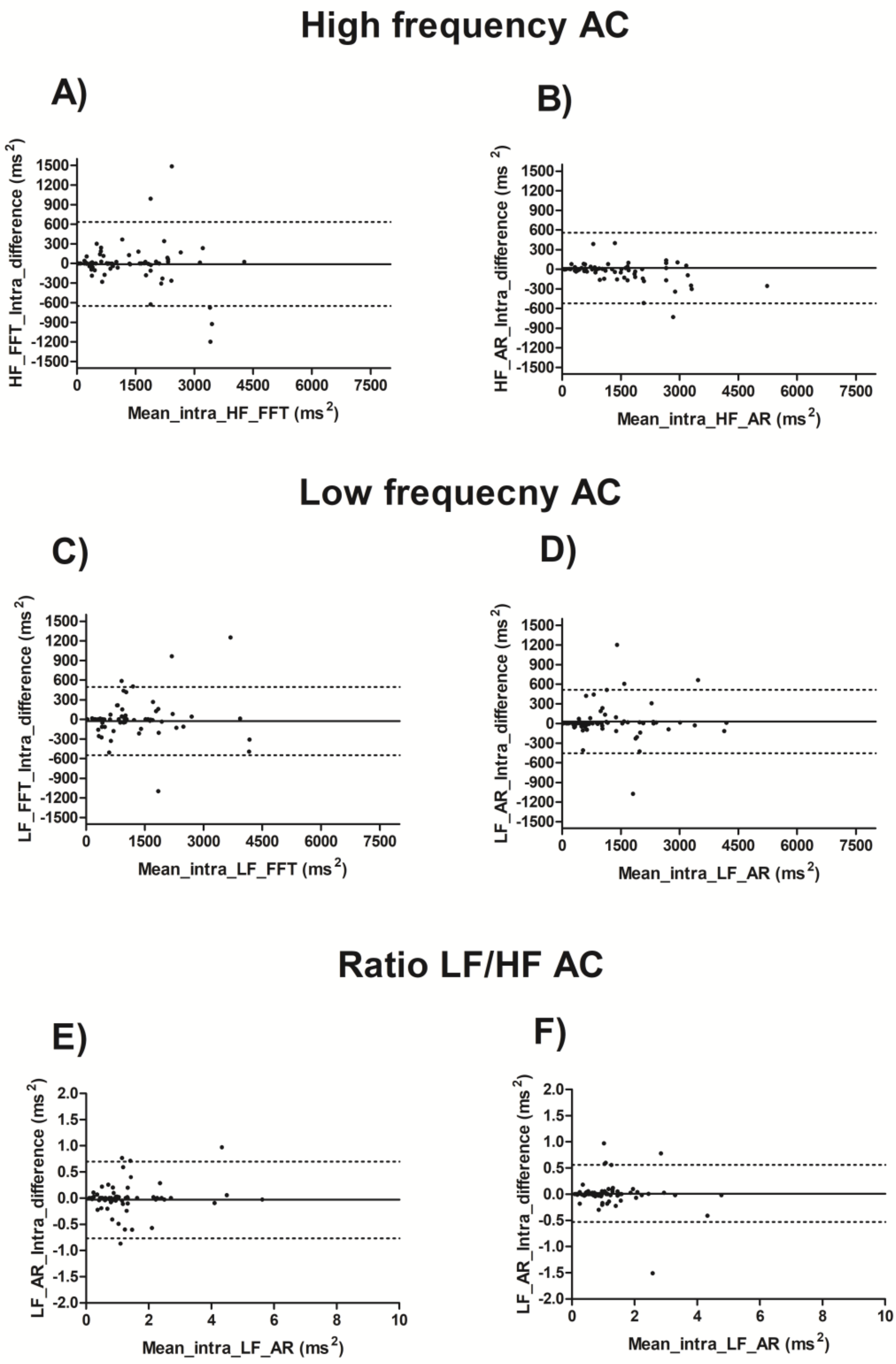

Figure S3. Bland-Altman plots for frequency-domain HRV parameters (intra-reliability) in young adults, ACTIBATE study (AC): panels A and B) are for high-frequency, panels C and D) low-frequency and, panels E and F) for ratio LF/HF with FFT and AR algorithms, respectively.

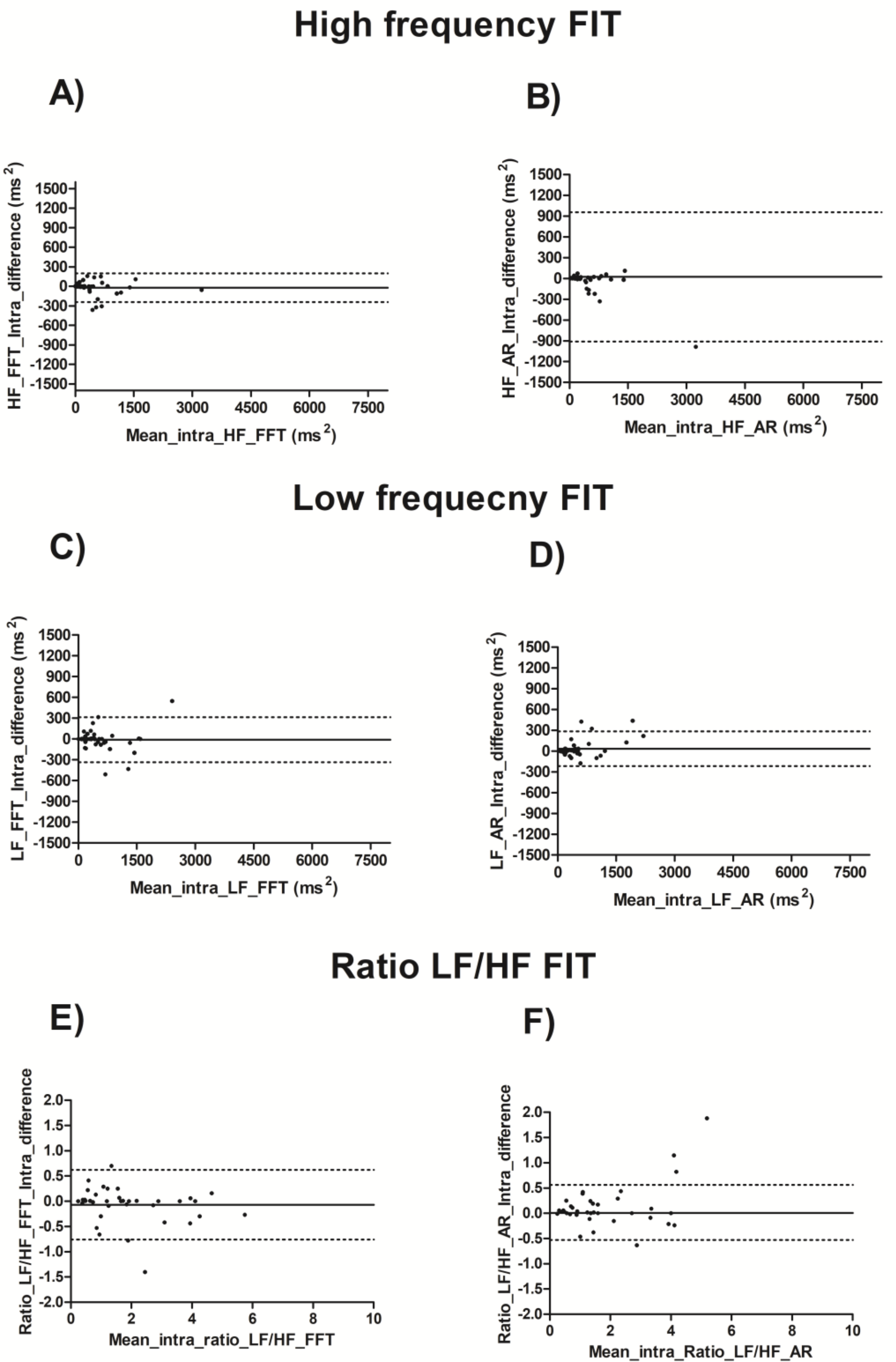

Figure S4. Bland-Altman plots for frequency-domain HRV parameters (intra-reliability) in middle-aged adults, FIT-AGEING study (FIT): panels A and B) are for high-frequency, panels C and D) low-frequency and, panels E and F) for ratio LF/HF with FFT and AR algorithms, respectively.

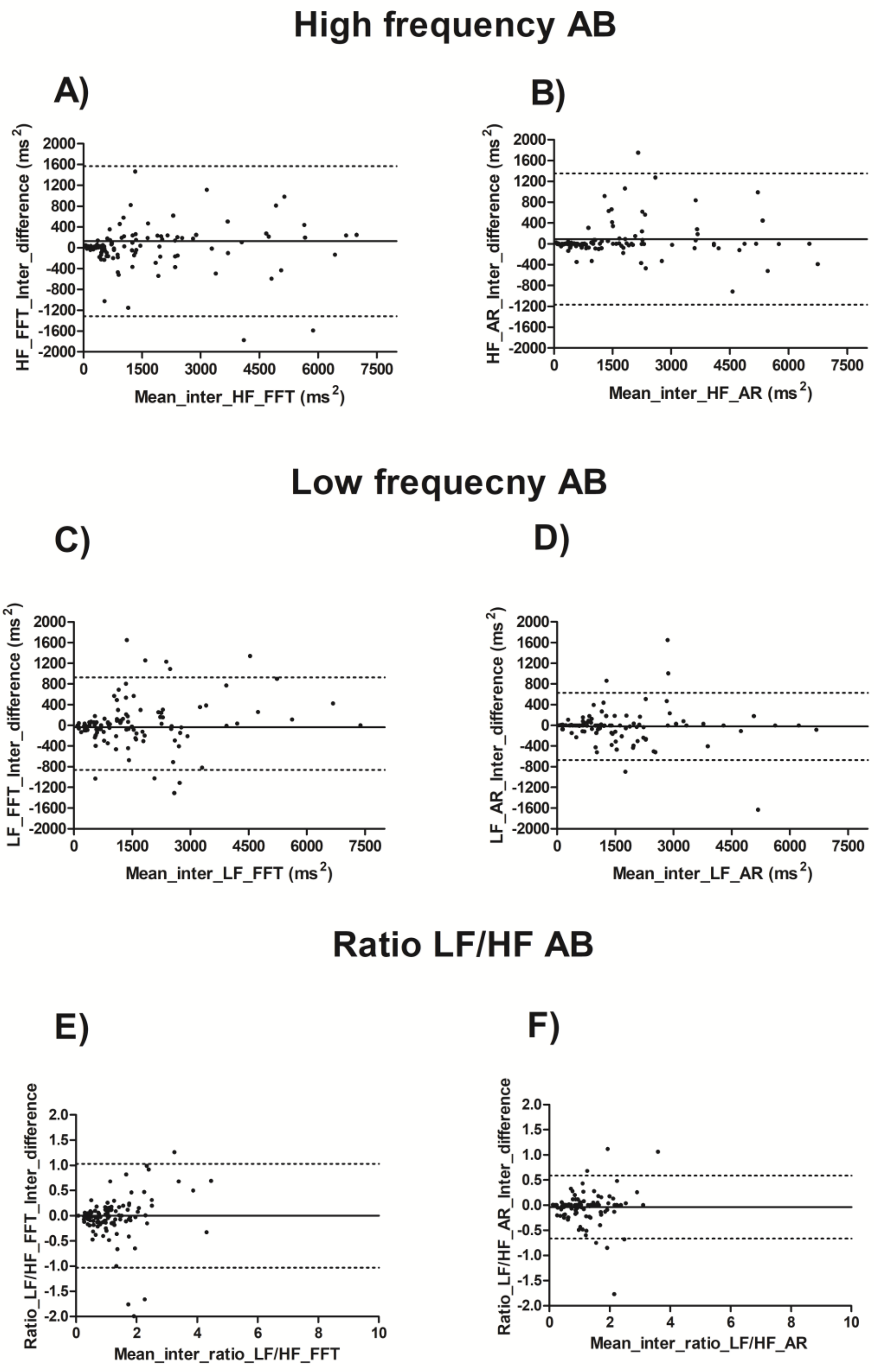

Figure S5. Bland-Altman plots for frequency-domain HRV parameters (inter-reliability) in middle-aged adults, ActiveBrains project (AB): panels A and B) are for high-frequency, panels C and D) low-frequency and, panels E and F) for ratio LF/HF with FFT and AR algorithms, respectively.

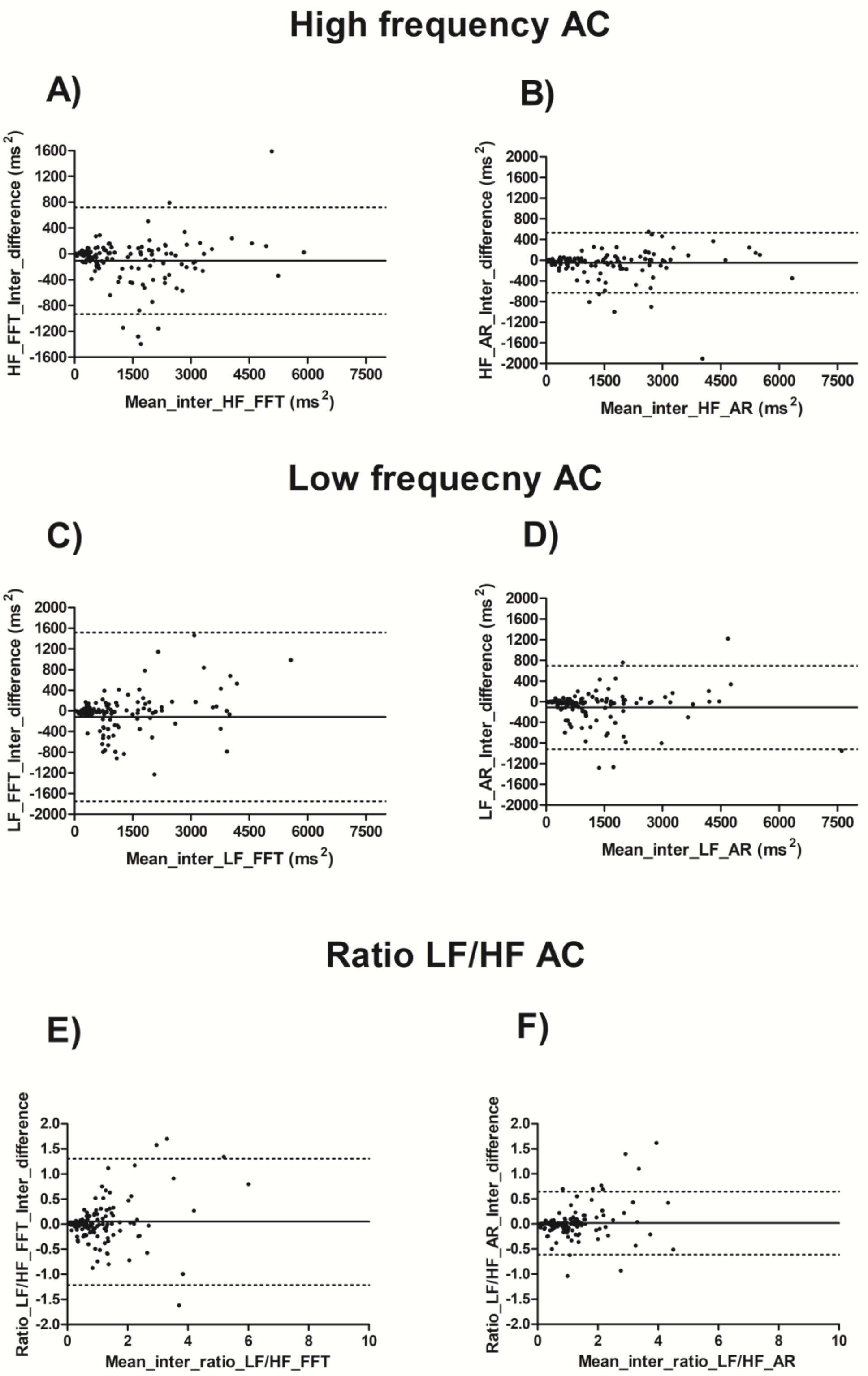

Figure S6. Bland-Altman plots for frequency-domain HRV parameters (inter-reliability) in middle-aged adults, ACTIBATE study (AC): panels A and B) are for high-frequency,

panels C and D) low-frequency and, panels E and F) for ratio LF/HF with FFT and AR algorithms, respectively.

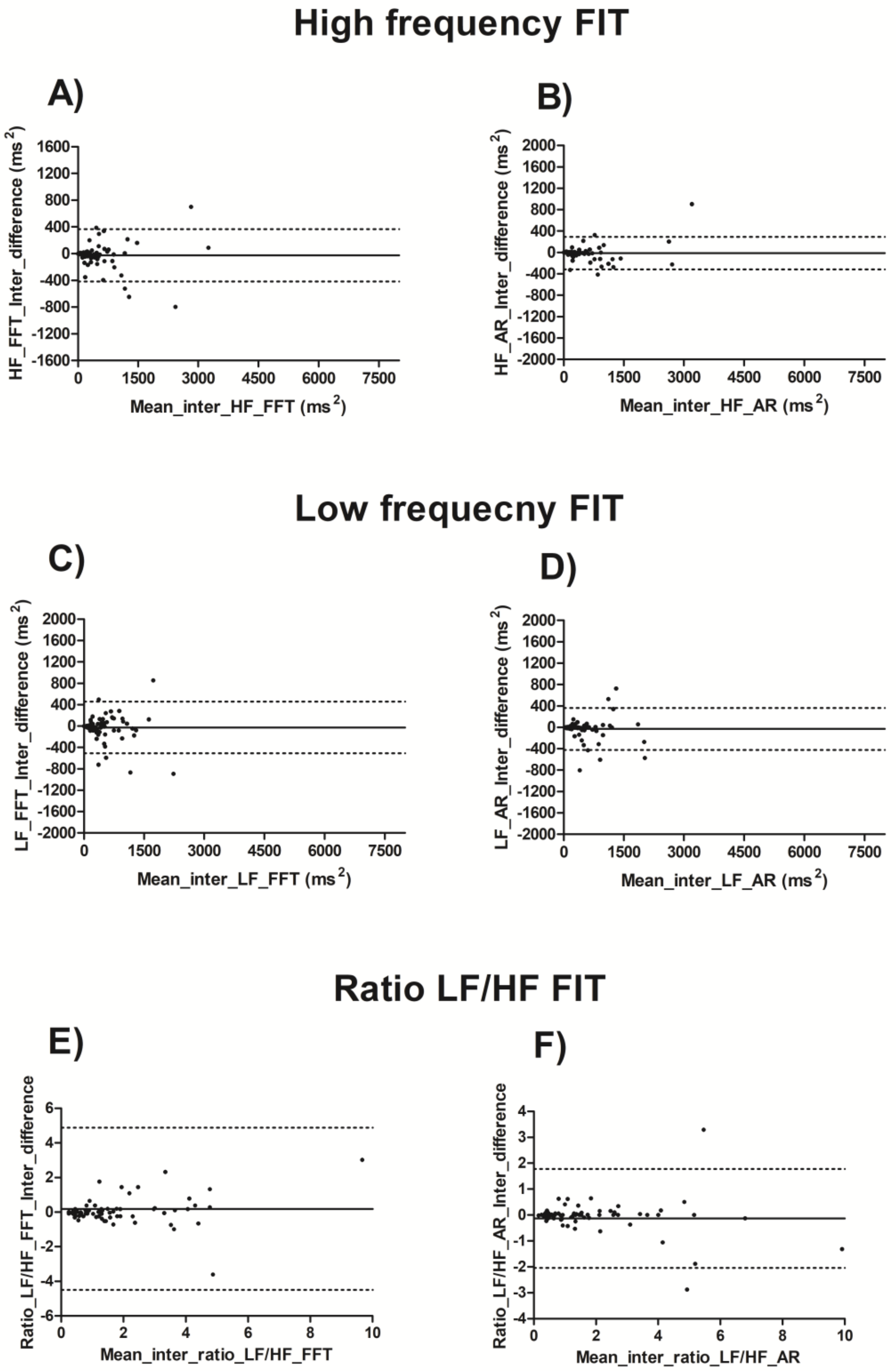

Figure S7. Bland-Altman plots for frequency-domain HRV parameters (inter-reliability) in middle-aged adults, FIT-AGEING study (FIT): panels A and B) are for high-frequency,

panels C and D) low-frequency and, panels E and F) for ratio LF/HF with FFT and AR algorithms, respectively.
